# Investigating Experimental and Computational Fluid Dynamics of 3D-Printed TPMS and Lattice Porous Structures

**DOI:** 10.3390/mi16080883

**Published:** 2025-07-29

**Authors:** Guru Varun Penubarthi, Kishore Bhaskar Suresh Babu, Senthilkumar Sundararaj, Shung Wen Kang

**Affiliations:** 1 Department of Mechanical and Electro-Mechanical Engineering, Tamkang University, New Taipei City 25137, Taiwan; 813375010@o365.tku.edu.tw; 2 Department of Aeronautics, Imperial College London, London SW7 2AZ, UK; kb724@ic.ac.uk; 3 Department of Aerospace Engineering, SRM Institute of Science and Technology, Kattankulathur, Chennai 603203, India; senthils7@srmist.edu.in

**Keywords:** additive manufacturing, triply periodic minimal surfaces (TPMS), contact angle, permeability, capillary performance

## Abstract

This study investigates the capillary performance and wetting behavior of SLA (Stereolithography) 3D-printed porous structures, focusing on TPMS (triply periodic minimal surfaces)-Gyroid, Octet, Diamond, and Isotruss lattice designs. High-speed imaging was used to analyze droplet interactions, including penetration, spreading, and contact angles, with 16 μL water droplets dropping from 30 mm at 0.77 m/s. Results showed variable contact angles, with Isotruss and Octet having higher angles, while Diamond faced measurement challenges due to surface roughness. Numerical simulations of TPMS-Gyroid of 2 mm^3^ unit cells validated the experimental results, and Diamond, Octet, and Isotruss structures were simulated. Capillary performance was assessed through deionized (DI) water weight–time (w-t) measurements, identifying that the TPMS-Gyroid structure performed adequately. Structures with 4 mm^3^ unit cells had low capillary performance, excluding them from permeability testing, whereas smaller 2 mm^3^ structures demonstrated capillary effects but had printability and cleaning issues. Permeability results indicated that Octet performed best, followed by Isotruss, Diamond, and TPMS-Gyroid. Findings emphasize unit cell size, beam thickness, and droplet positioning as key factors in optimizing fluid dynamics for cooling, filtration, and fluid management.

## 1. Introduction

Heat pipes are essential in managing heat across a range of applications, such as in electronics like laptops, desktops, and LED (Light-Emitting Diode) lights, where they prevent overheating and maintain optimal performance. They also play a critical role in spacecraft thermal management, protecting sensitive electronics from temperature fluctuations. In HVAC (Heating, Ventilation, and Air Conditioning) systems, power plant cooling, high-performance CPUs and GPUs, and heat pipes are indispensable for ensuring efficient thermal control. The advancement of 3D printing has facilitated the precise creation of complex geometries used in aerospace, biomedical engineering, and energy systems. Specifically, 3D printing allows us to produce porous structures that aid in fluid handling processes, such as liquid dispersion and capillary action.

This study investigates the fluid dynamics and permeability of 3D-printed porous structures to deepen our understanding of capillary-driven phenomena and optimize design across various industries. Heat pipe performance is significantly influenced by fluid properties like wettability, permeability, and porosity, with the contact angle playing a key role in heat transfer. The contact angle, which measures fluid wettability on surfaces, directly affects heat transfer efficiency. Lower contact angles indicate better wettability, enhancing heat transfer. In applications such as inkjet printing and membrane technology, the contact angle governs fluid spreading and penetration. While research has focused on substrate pore size and liquid properties, no studies have fully explored the effects of absorption, drop size, and surface tension on contact angles in TPMS structures.

Recent advances in triply periodic minimal surface (TPMS) structures and porous media have enabled significant progress in thermal management systems. Guddati et al. (2019) [1] reviewed additive-manufactured porous architectures for thermal applications, emphasizing the importance of surface area density and geometric trade-offs. Oh et al. (2023) [2] further demonstrated how TPMS morphology optimization can enhance flow characteristics in compact heat exchangers. In the realm of wetting behavior on porous substrates, Krainer and Hirn (2021) [3] showed that pore geometry and droplet size jointly influence contact angle evolution, while Han et al. (2022) [4] introduced an open-source, low-cost contact angle analyzer for high-resolution dynamic wetting measurements. Complementing these efforts, Kyrloglou et al. (2024) [5] validated volume-of-fluid (VOF) simulations of droplet dispersion in mesoporous membranes, reinforcing the predictive accuracy of such models. Studies on capillary and permeability behavior by Jafari et al. (2018) [6] revealed how metal 3D-printed wick geometries influence capillary rise, and Sandoval et al. (2023) [7] compared permeability test methods in porous materials, providing valuable experimental insight.

Building on this foundation, our study presents the first combined experimental–numerical analysis of dynamic droplet behavior across four distinct 3D-printed structures—Gyroid, Octet, Diamond, and Isotruss—achieving <6% error through synchronized high-speed imaging and VOF simulations. Additionally, we quantitatively assess capillary rise and constant head permeability across unit-cell sizes (2–4 mm^3^), revealing trade-offs between wettability, pore geometry, and printability. The findings offer practical design guidance for optimizing additive manufacturing parameters, such as beam thickness and unit-cell geometry, tailored to enhance thermal performance in heat pipes and related phase-change cooling devices.

Recent developments in triply periodic minimal surface (TPMS) architectures have demonstrated their exceptional potential for improving heat transfer, fluid permeability, and capillary-driven absorption [8,9,10,11]. Zhao et al. [12] showed that hybrid TPMS wicks significantly enhance fluid return while lowering overall thermal resistance, validating their suitability for high-performance thermal systems. Similarly, Saghir and Kilic [13] provided a comprehensive analysis of forced convection and nanofluid heat transfer through TPMS structures, reinforcing both the reliability of permeability measurements and the advantages of additive manufacturing for wick fabrication.

Building on this extensive experimental and analytical groundwork, the present study employs high-fidelity numerical simulations—integrating volume-of-fluid (VOF) techniques with dynamic wetting models—to resolve multi-fluid transport and contact-angle evolution within 3D-printed TPMS lattices. Our aim is to translate these insights into design guidelines for applications spanning heat exchangers, loop-heat-pipe wicks, and microfluidic wicking devices.

## 2. Design and Manufacturing

### 2.1. Designing of Porous Medium

**Figure 1:**
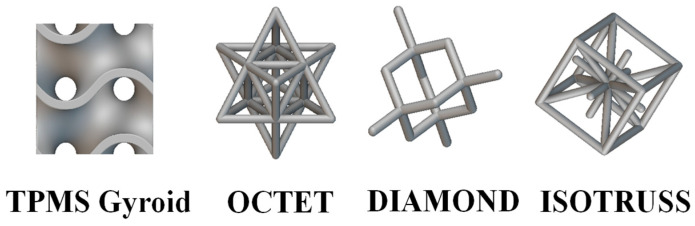
Various unit cells used in this study.

Recent advancements in additive manufacturing (AM) have accelerated the development of TPMSs for heat exchange (HX) applications. While TPMS structures offer high surface area density, significantly enhancing heat exchange capabilities, further design modifications are necessary to make them suitable for heat pipe (HP) application [13]. Building on these findings, our current study focuses on TPMS structures, specifically the TPMS Gyroid, which aims to outperform traditional unit cells due to its unique geometric properties. Four models were designed using nTopology (v4.19.2), as shown in Figure 1.

**Figure 2:**
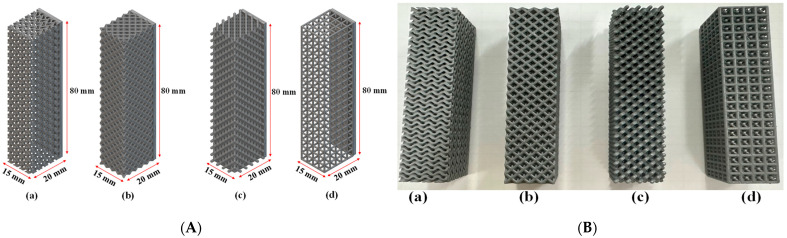
(**A**) Porous structures designed for contact angle measurement test: a—TPMS Gyroid, b—Octet, c—Diamond, and d—Isotruss; (**B**) 3D-printed porous structures: a—TPMS Gyroid, b—Octet, c—Diamond, and d—Isotruss.

We refined our methods to re-evaluate Octet, diamond, and Isotruss structures under updated conditions, improving the accuracy of permeability and capillary performance measurements. This reassessment deepens our understanding and advances TPMS Gyroid applications in additive manufacturing and thermal management. The surface area-to-volume (SA/V) ratio was examined as a key factor affecting heat and mass transfer efficiency, revealing differences based on geometry and unit cell size. Contact angle measurements were conducted on samples sized 80 mm in height, 20 mm in width, and 15 mm in length (Table 1, Figure 2A) to ensure consistent, reliable results.

**Figure 3:**
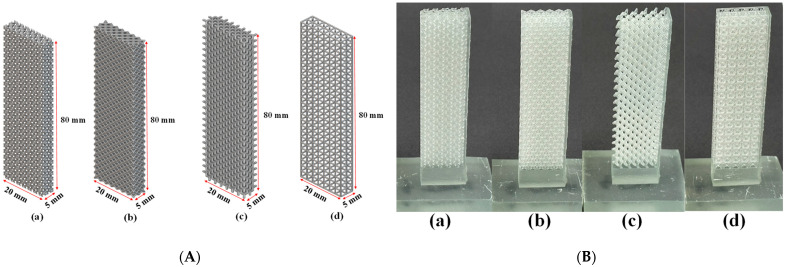
(**A**) Porous structures designed for capillary performance and permeability tests: a—TPMS Gyroid, b—Octet, c—Diamond, and d—Isotruss; (**B**) 3D-printed porous structures: a—TPMS Gyroid, b—Octet, c—Diamond, and d—Isotruss.

In our initial experiments on capillary performance and permeability, we used larger porous media samples as outlined in Table 1. However, their complexity posed challenges for 3D printing. To address this, we reduced the sample width to one-third of its original size, making the printing process more manageable while maintaining structural integrity. Despite the size reduction, we kept the porosity around 50% and adjusted the beam thickness, as shown in Table 2, to preserve the desired structural properties. These modifications enabled more efficient production of test samples without compromising accuracy or relevance. The updated sample dimensions are shown in Figure 3A, illustrating the changes made to accommodate the new size. This approach allowed for a more practical production process while ensuring that the test samples remained effective and true to the original design.

**Figure 4:**
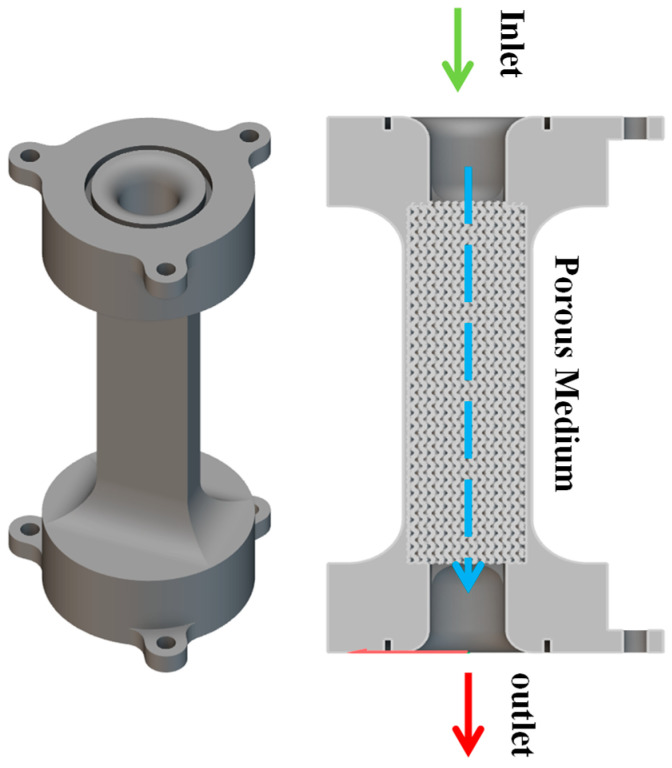
Schematic diagram of permeability experiment.

A porous structure was designed based on parameters in Table 2 to assess its permeability, as depicted in Figure 4. Water flows through the structure from an inlet to an outlet, with a fixed-height container at the top ensuring a steady supply and an overflow mechanism preventing excess.

### 2.2. Manufacturing of Porous Medium

The lattice structures were fabricated using the Phrozen Mega 8K S 3D printer, which features high-resolution LCD (liquid crystal display) technology. This method offers the precision necessary for reproducing intricate geometries, making it well-suited for complex lattice designs. The printer operates on the SLA (Stereolithography) principle, where photopolymer resin is solidified layer by layer through UV (Ultraviolet) radiation with a layer thickness ranging from 0.01 to 0.3 mm and exposure time per layer ranging from 8–15 s, enabling the creation of highly detailed and dimensionally accurate structures. After printing, the samples underwent ultrasonic cleaning in ethanol, followed by UV post-curing for 10–15 min to ensure complete polymerization and structural integrity.

LCD-based 3D printing was selected for this study due to its cost-effectiveness, faster printing time, and ease of iterations, allowing for rapid corrections and improvements during the design and prototyping phase. Although LCD printing has certain limitations compared to other methods, it proved to be an effective and reliable solution within the scope of this research. This study focuses exclusively on fluid dynamics and does not involve heat transfer analysis, thereby eliminating the immediate need for thermally conductive materials. Consequently, resin-based porous structures were sufficient to meet the experimental objectives, making metal 3D printing unnecessary at this stage. It is important to note that this work represents a preliminary investigation. In future phases, the study will be extended to include boiling heat transfer and high-temperature testing using metal 3D-printed samples. These advanced samples will be fabricated using the Tongtai AMP-160 Metal 3D printer (Tamkang University, Tamsui, Taiwan), which is currently available and suitable for producing high-precision stainless steel porous structures. This progression will allow for a more comprehensive evaluation of the porous geometries under thermal loads and in realistic application environments.

Different types of resins were carefully selected based on the specific needs of each experiment. For the contact angle study, TR300 resin was used due to its high thermal resistance and surface stability, providing a consistent platform for accurate droplet measurement. For the capillary rise experiments, Aqua Clear resin was selected for its transparency, which enabled clear visual tracking of the fluid movement within the porous structure. Lastly, for the permeability tests, Hyperfine Blue resin was selected for this experiment due to its fast-curing capability, which is particularly advantageous for structures enclosed within a confined domain where thorough cleaning and post-curing are challenging. This resin also provides high dimensional accuracy and consistent mechanical strength, enhancing printing efficiency while maintaining structural integrity and fidelity. The resin selection was crucial to achieving the objectives of each experiment while maintaining high-resolution lattice integrity. All manufacturing was carried out at Tamkang University, Tamsui, Taiwan, using the equipment currently available.

## 3. Experimental Setup

### 3.1. Contact Angle Measurement

**Figure 5:**
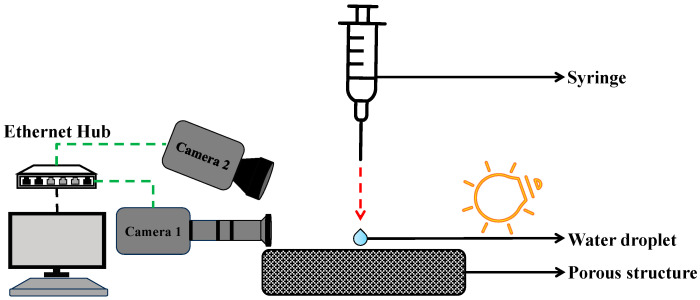
Schematic diagram of contact angle measurement experimental setup.

This experiment uses a custom setup for precise contact angle measurements, shown in Figure 5. It integrates two synchronized high-speed cameras with microscopic lenses to capture dynamic wettability data. A 16 μL water droplet, approximately 1.529 mm in diameter, was released from a height of 30 mm onto the sample surface. The droplet size was intentionally chosen to be larger than the pore radius of all structures to ensure consistent interaction across different geometries. Controlling droplet mass was a challenge, addressed with a KDS high-pressure water pump for accurate deposition. Tests ensured repeatability of droplet mass. “ImageJ (version 1.54g)” is public domain software used for image processing of high-speed videos frame-by-frame to measure contact angles, employing advanced image processing to detect droplet edges and calculate angles accurately under varying conditions [3].

The contact angle is measured on both the right and left sides of the droplet, and the average value is calculated from these measurements. Among multiple tests, the representative average is selected, typically falling between the range of results—to determine the mean contact angle. This approach allows us to quantify the measurement variability by calculating the error margin based on the repeated test outcomes.

### 3.2. Capillary Performance Test

**Figure 6:**
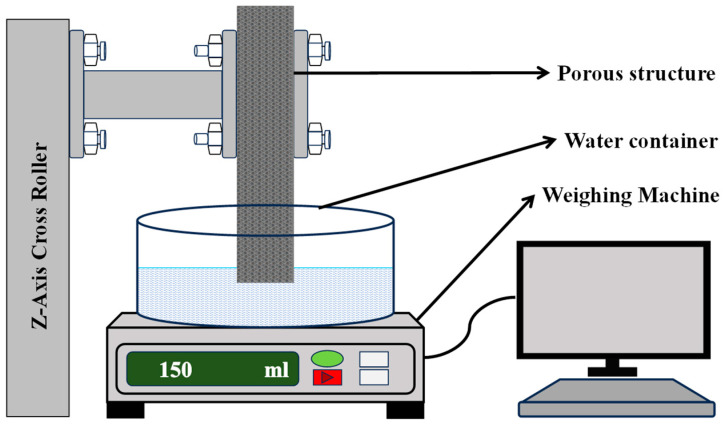
Schematic diagram of capillary performance test experimental setup.

This experiment employed a specialized setup, as shown in Figure 6, featuring a precision weighing machine for accurate sample mass estimation. Its software interface allows for quick parameter entry and precise data collection. A *z*-axis controller stage ensured stable vertical movement and sample positioning, maintaining consistent results. A custom holder secured samples during measurements, minimizing disturbance and enabling efficient loading and unloading. The combination of precise measurement tools, controlled movement, and custom holders enhanced the accuracy and reliability of the experiment [5].

### 3.3. Constant-Head Permeability Test

**Figure 7:**
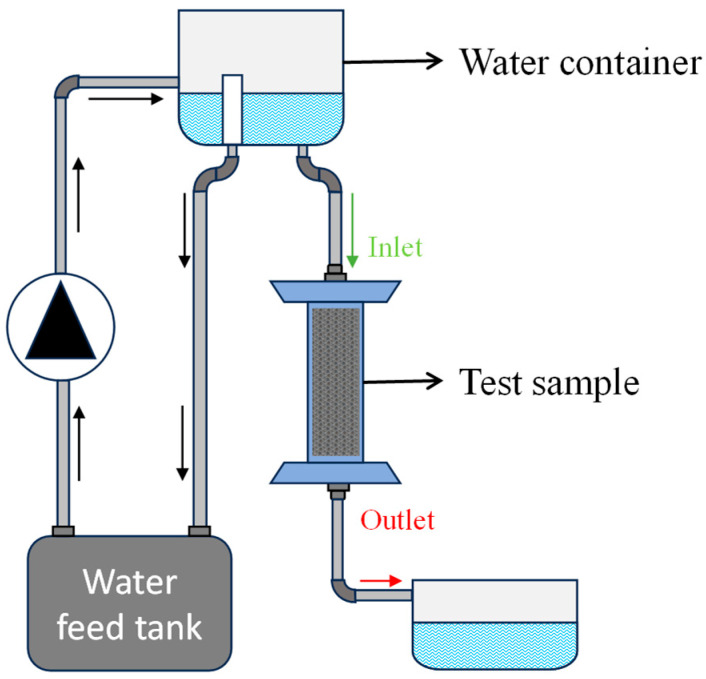
Schematic diagram of constant-head permeability experimental setup.

The constant head permeability experiment used the constant head method to measure permeability in porous structures, as shown in Figure 7. This technique maintains a steady hydraulic head, ideal for high-permeability materials like sands. Water flows from a higher to a lower elevation, with the height difference constant. The flow rate through the structure was recorded, and permeability was calculated using Darcy’s Law, factoring in flow rate, cross-sectional area, fluid viscosity, and hydraulic gradient. As seen in Figure 7, the setup includes a porous structure, water inlet, bottom outlet, and an overflow mechanism to stabilize water levels [6].

The permeability of a porous medium is typically calculated using models that correlate the microstructural characteristics of the medium, such as pore size, shape, and distribution, to its permeability. A widely used method for this calculation is Darcy’s Law. Darcy’s law (1) describes the flow of fluid through a porous medium.

Rearranging Darcy’s law to solve permeability, *k*
  (1) $$Q=-k\times A\times \frac{∆h}{L}$$
Determine the volume of water (*V*) collected as follows:
  (2) $$k=\frac{Q\times L}{A\times ∆h}$$
For water at room temperature, *ρ* ≈ 1000 kg/m^3^.
  (3) $$V=\frac{W}{\rho }$$
Substituting values to find volumetric flow rate, Q, as follows:
  (4) $$Q=\frac{V}{t}$$

Calculate *k* from the above Darcy’s Law, which gives the permeability of the porous medium.

where
•*Q*=Volumetric flow rate(m^3^/s)•*k*=Permeability(m^2^)•*A*=Cross-sectional area(m^2^)•∆h=Pressure head difference (m)•*L*=Length or thickness of porous sample(m)•*V*=Volume of fluid collected(m^3^)•*W*=Mass (or weight) of fluid collected(kg)•*ρ*=Density of the fluid (water)(kg/m^3^)•*t*=Time(s)

## 4. Numerical Setup of Contact Angle

**Figure 8:**
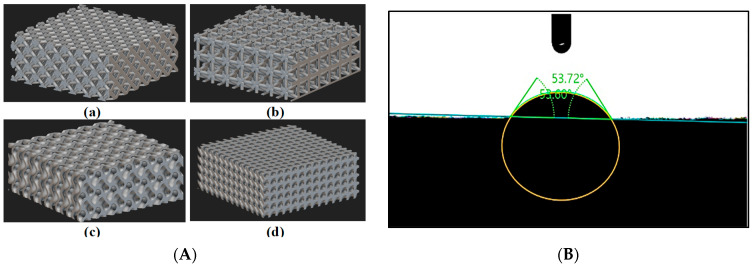
(**A**) Porous structures designed using nTopology: a—Octet, b—Isotruss, c—TPMS-Gyroid, and d—Diamond; (**B**) static contact angle of the solid 3D-printed flat plate measured by dpiMAX goniometer.

Four models were created, namely TPMS Gyroid, Diamond, Octet, and Isotruss, with a unit cell size of 2 × 2 × 2 mm^3^ and dimensions of 10 × 10 × 3 mm^3^, using commercial implicit CAD modeling software nTopology. The modeling equations used to create the TPMS structures are detailed in Table 3 below. The CAD model, designed using nTopology, is shown in Figure 8A.

The static contact angle needs to be determined before proceeding with the simulation of different structures. Therefore, a 3D-printed flat plate was fabricated using the same resin as the test samples used in the experiments, and a water droplet was placed on its surface. The measured static contact angle was 53.72°. The figure below shows the experimental results.

To validate the simulation’s accuracy, in this experiment, distilled water droplets of 16 microliters were deposited on the porous TPMS-Gyroid structure from a height of 30 mm. The machine employed for this task is equipped with a high-resolution camera and precision syringe control. These features are essential for capturing the evolution of the droplets that are in contact with the surface and ensuring accurate volume repeatability. The dynamic contact angle was measured with the help of the elliptical fit method from the goniometer (Figure 8B), and the experimental setup is shown in Figure 5.

**Figure 9:**
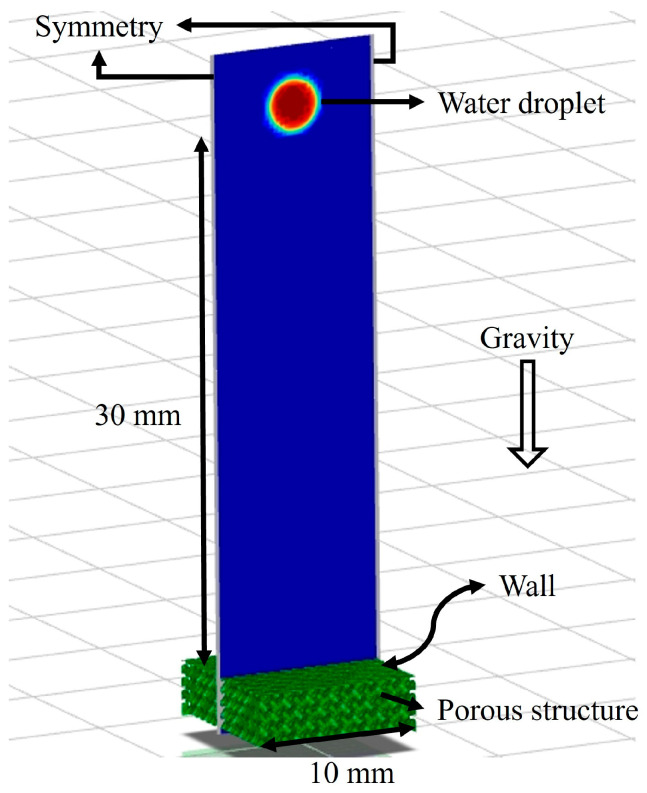
Fluid domain with applied boundary conditions in the numerical model.

The boundary conditions and the geometry for validation are given. The fluid domain with applied boundary conditions is shown in Figure 9. The following assumptions are used to simulate the drop dynamics:
Flow Type:Transient, LaminarGravity:9.81 $m/s^{2}$
Height at Which the Water was Dropped:30 mmMultiphase Model:VOFVolume:16 μLDroplet Diameter:1.529 mmDroplet Fluid Material:Water

The VOF (Volume of Fluid) with a transient, multiphase model was used. The tracking interface between different phases is achieved by solving the continuity equation for the volume fraction of phases. For the *q*th phase, this equation has the following form [14]:

(5) $$\frac{1}{\rho_{q}}\;[\frac{\partial }{\partial t}\;\left(\alpha_{q}\rho_{q}\right)+\;\nabla \cdot \left(\alpha_{q}\rho_{q}{\dot{v}}_{q}\right)=\;s_{\alpha_{q}}+\;\sum_{p=1}^{n}\left({\dot{m}}_{pq}{\dot{m}}_{qp}\right)]$$

In which, for *m_qp_* mass transfer from phase 1 to phase 2, *m_pq_* is vice versa, and the source term is by default zero. However, as there is no phase change occurring in this study, *m_qp_* and *m_pq_* are equal to zero.

The volume fraction equation is solved by explicit time discretization, as follows:

(6) $$\frac{\alpha_{q}^{n+1}\rho_{q}^{n+1}-{\alpha_{q}^{n}\rho }_{q}^{n}}{∆t}V+\;\sum_{f}\left(\rho_{q}U_{f}^{n}\alpha_{q,f}^{n}\right)=[\sum_{p=1}^{n}{\dot{(m}}_{pq}-{\dot{m}}_{qp})+S_{\alpha_{q}}]V$$

where
•α=Volume fraction of the secondary fluid •v=Velocity vector (m/s) •t=Time (s)

In each computational cell, the volume fraction of each phase is tracked as follows:
•α = 1=Cell is full of the primary fluid•α = 0=Cell is full of the secondary fluid•0 < α < 1=Cell contains the interface 


A geometric reconstruction scheme was used for the interface capture of the two phases of fluids. A single momentum equation is solved throughout the domain, and the resulting velocity term is shared among the phases. The momentum term shown below is dependent on the volume fractions of all the phases:

(7) $$\frac{\partial }{\partial t}\left(\rho \bar{v}\right)+\nabla \cdot \left(\rho \bar{v}\bar{v}\right)=-\nabla p+\nabla \cdot \left[\mu \left(\nabla \bar{v}+\nabla {\bar{v}}^{T}\right)\right]+\rho \bar{g}+F$$

where
•*ρ*=Volume-fraction-weighted local density (kg/m^3^) •*μ*=Volume-fraction-weighted dynamic viscosity (kg/m.s) •*p*=Pressure (N/m^2^) •*g*=Gravity vector (m/s^2^) •*F*=Surface tension force per unit volume (N/m) 

The surface tension between air and water was 0.072 N/m, using the continuum surface force model [15] to maintain consistent contact angles.

**Figure 10:**
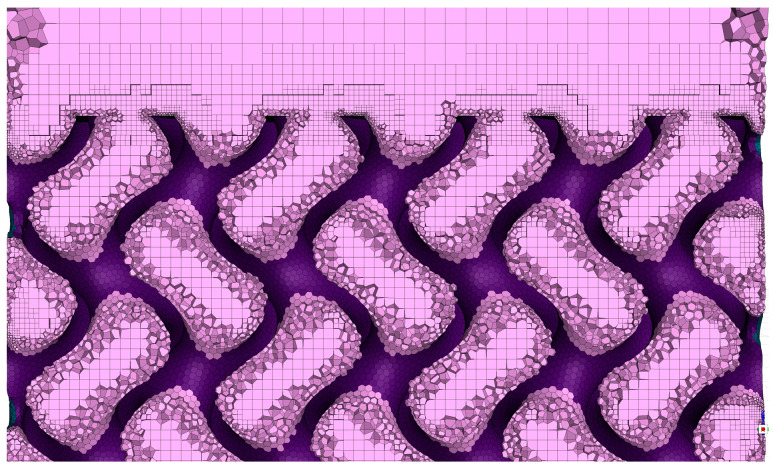
Polyhexa mesh for Gyroid structure.

After simulating the TPMS-Gyroid structure, the structure was then replaced with Diamond, Isotruss, and Octet structures (Figure 8A) while maintaining the same boundary conditions and fluid domain. Droplet dynamics were captured and contact angles were measured with a goniometer. The fluid domain was finely meshed, with a max cell size of 0.2 mm, refined near the surface (Figure 10).

## 5. Results and Discussion

### 5.1. Contact Angle Measurement Test

#### 5.1.1. Experimental Results

**Figure 11:**
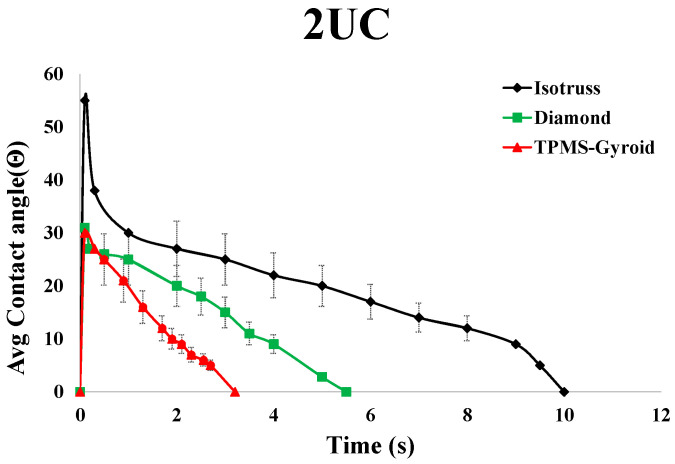

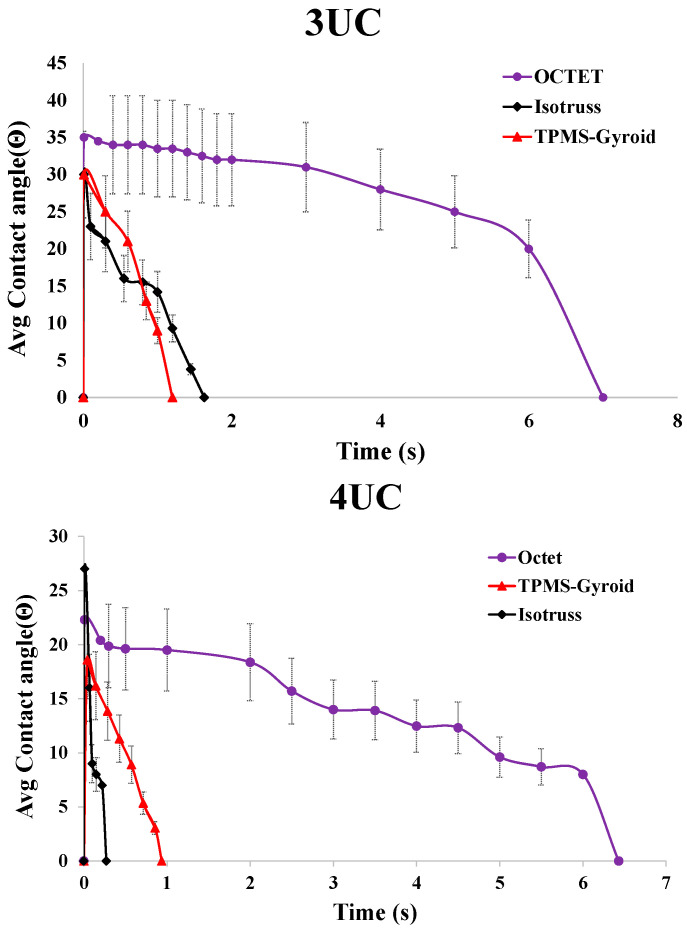
Contact angle measurement test results of 2, 3, and 4 mm^3^ unit cell structures.

The dynamic contact angle test tracks liquid droplet behavior on porous surfaces over time, revealing insights into wetting, penetration, and contact angle as shown in Figure 11. Larger pores (4 mm^3^) allow quicker fluid penetration and smaller contact angles, while smaller pores (2 mm^3^) result in slower penetration and larger angles, with the 3 mm^3^ unit showing intermediate values. Experimental results for 2 mm^3^ show TPMS-Gyroid required less time than other structures, with Isotruss taking the longest. For 3 mm^3^, TPMS-Gyroid performed best, while Isotruss took more time than TPMS-Gyroid and Diamond. For 4 mm^3^, Isotruss performed best, followed by TPMS-Gyroid, with Octet taking the longest time. Results for the 2 mm^3^ Octet structure were not obtained due to challenges during printing and cleaning and insufficient fluid flow. For the 3 and 4 mm^3^ Diamond structures, the droplet penetrated too rapidly to accurately capture the contact angle.

#### 5.1.2. Repeatability and Uncertainty Analysis

**Figure 12:**
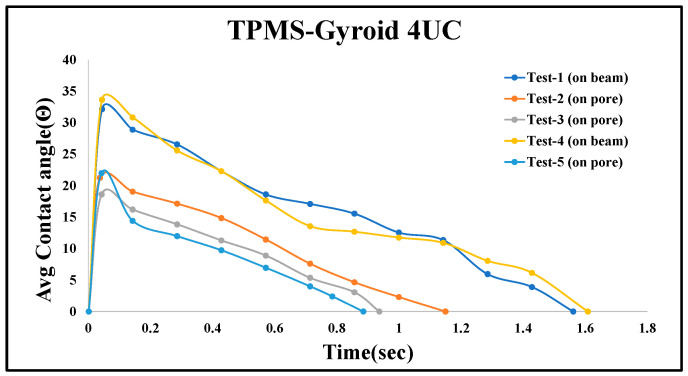
Average dynamic contact angle measurements at multiple locations on a 4-unit-cell TPMS-Gyroid structure.

To assess repeatability and highlight experimental variability, five contact angle tests were conducted on the TPMS-Gyroid structure with a 4 mm^3^ unit cell. Figure 12 illustrates the consistency and influence of droplet placement. While all tests followed a similar trend, variations in penetration time were observed based on where the droplet landed. Tests 1 and 4, in which droplets were placed on the beam surface of the structure, showed slower absorption (1.62 and 1.56 s) due to initial spreading and delayed entry. In contrast, Tests 2, 3, and 5, where droplets were placed directly over the pores, penetrated significantly faster (1.15, 0.93, and 0.88 s). This emphasizes that wettability behavior is highly dependent on surface interaction zones. Although similar trends were observed in other structures, only one representative graph is shown for clarity. Future studies will increase the sample size and employ controlled placement strategies to enhance statistical confidence.

Contact angle measurements were performed multiple times, with angles recorded on both the left and right sides of each droplet. The average contact angle was computed for each droplet to minimize side-dependent bias. Repeated measurements allowed statistical evaluation of variability, quantified by the standard deviation. The overall mean contact angle $\bar{\theta }$ and its standard deviation *σ* were calculated as follows:

(8) $$\bar{\theta }=\frac{1}{N}\sum_{i=1}^{N}\theta_{i},\;\sigma =\sqrt{\frac{1}{N-1}\sum_{i=1}^{N}{\left(\theta_{i}-\bar{\theta }\right)}^{2}}$$

where
•$\theta_{i}$=Average contact angle •$\bar{\theta }$=Mean contact angle across all measurements •σ=Standard deviation of the measured contact angles •N=Total number of valid droplet measurements used in the analysis •$E_{exp}$=Experimental error (%)•$E_{int}$=Instrumental uncertainty (0.2%) •$E_{total}$=Total uncertainty (%)

The experimental error percentage was derived from the relative standard deviation, as follows:

(9) $$E_{exp}=\left(\frac{\sigma }{\bar{\theta }}\right)\times 100\%\;$$

The instrument error of the OCA software, specified as 0.2%, was combined with the experimental error using the root-sum-square method, as follows:

(10) $$E_{total}=\sqrt{E_{int}^{2}+E_{exp}^{2}}$$

This combined uncertainty captures both the limitations of the instrument and the variability from the repeated tests.

In the initial stage, the droplet shape is clear and stable, allowing accurate angle detection with minimal error. Toward the end, as the droplet depletes and penetrates the structure, its boundaries become unclear. The software struggles to detect the droplet accurately, and surface wetness adds noise, leading to higher measurement uncertainty in the final stage. Based on the full dataset of repeated measurements for the structure, the overall uncertainty was calculated to be approximately 19.4%, accounting for both experimental scatter and instrument precision.

#### 5.1.3. Numerical Study Results

**Figure 13:**
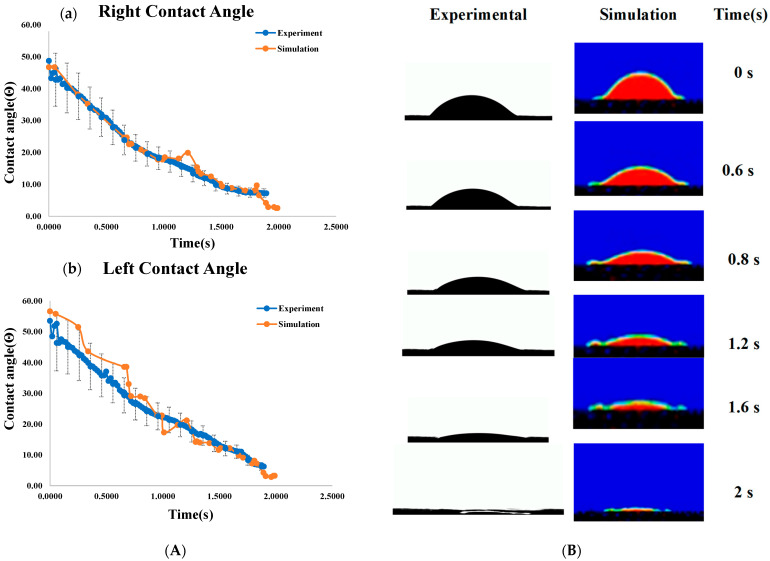
(**A**) Comparison of dynamic contact angles from experiments and simulations for the TPMS-Gyroid structure: a—right angle, b—left angle; (**B**) comparison of water droplet evolution of TPMS-Gyroid structure results from experiment and numerical simulation.

**Figure 14:**
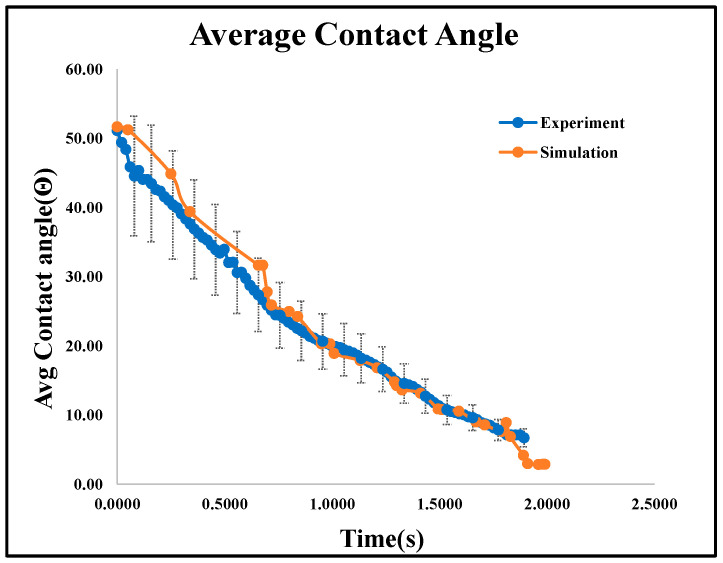
Average dynamic contact angle comparison of TPMS-Gyroid structure results from experiment and numerical simulation.

The right, left, and average dynamic contact angles from both experimental and simulated data are plotted in Figure 13A and Figure 14. Identical boundary conditions were applied, including a droplet volume of 16 μL, a drop height of 30 mm, and a Gyroid unit cell size of 2 × 2 × 2 mm^3^.

**Figure 15:**
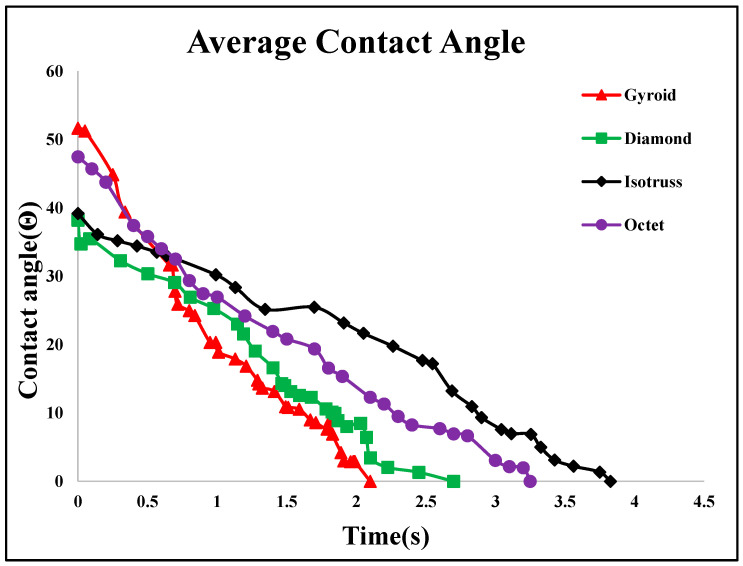
Numerically determined contact angles of all four structures.

The dynamic contact angles for both sides show minimal variation, confirming consistency across measurement methods. Experimental and numerical analyses recorded water droplet dispersion at various time intervals, as shown in Figure 13B, showing less than 6% error in comparison. The small discrepancy is due to printing thickness and roughness differences. Through the simulation results, it was found that all four structures exhibit a contact angle in the range of 50° to 30°, as shown in Figure 15. It shows the average dynamic contact angle for different structures. With a contact angle range of 52° to 0° and a penetration time of 2 s, the gyroid structure has the quickest fluid movement measured. The high initial contact angle suggests a surface that resists wetting, but rapid penetration shows that the droplet quickly wets the surface. By this phenomenon, a refrigerant can rapidly cover and transport heat over the evaporator part of heat pipes, making it ideal for fast heat transmission.

### 5.2. Capillary Performance Test Results

**Figure 16:**
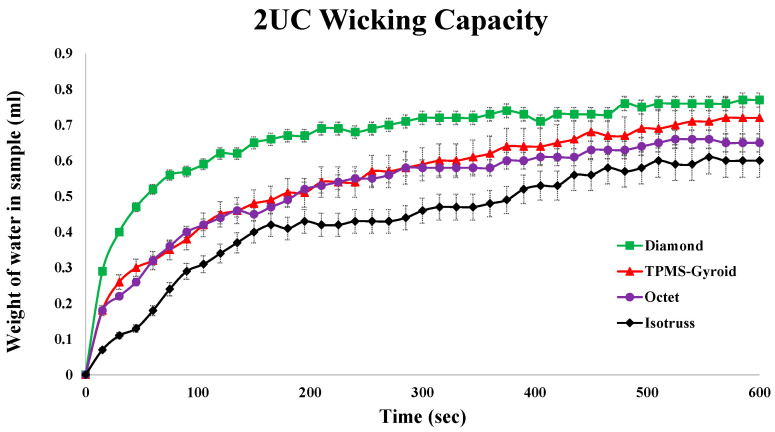

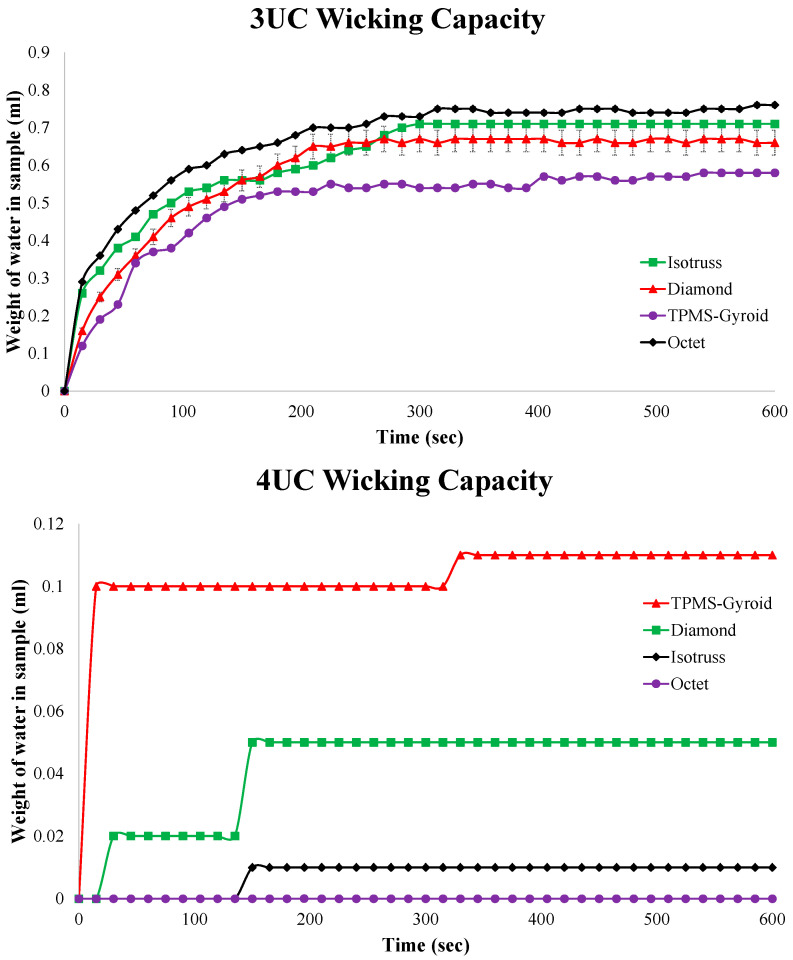
Capillary performance test results of 2, 3, and 4 mm^3^ unit cell structures.

This study evaluates capillary performance in 3D-printed porous media with unit cell sizes of 2 mm^3^, 3 mm^3^, and 4 mm^3^, as described in Table 2. Samples, with 50% porosity, were submerged in 150 mL of DI water, and weight changes were measured over 600 s. Figure 16 highlights how unit cell size affects wicking rates.

The Diamond structure showed the best performance at 2 mm^3^, followed by TPMS-Gyroid, Octet, and Isotruss. At 3 mm^3^, Isotruss outperformed the others, while at 4 mm^3^, all structures showed poor capillary performance due to larger pore radii.

An instrumental uncertainty of ±0.1 g was associated with the weighing balance. Additionally, three repeat tests were conducted for each structure, and the average relative error was calculated to represent the variability. Based on this, error bars were added to the graphs to reflect both instrument precision and repeatability.

### 5.3. Constant Head Permeability Test

The constant head permeability test was conducted on porous media with a 3 mm^3^ unit cell size. Due to printing challenges, 2 mm^3^ structures were not tested, and 4 mm^3^ structures were excluded after failing preliminary capillary tests. Table 4 presents permeability values (k) for each unit cell structure, along with their averages. From results obtained, the TPMS-Gyroid structure exhibited the highest average permeability (0.0185 m/s), indicating superior fluid flow capacity. The Diamond and Isotruss structures showed intermediate permeability values of 0.0182 m/s and 0.0167 m/s, respectively. The Octet structure had the lowest average permeability (0.0089 m/s), suggesting the least fluid flow capability. However, in terms of practical performance for heat pipe applications, the Octet structure performed best, followed by Isotruss, Diamond, and TPMS-Gyroid. This suggests that factors beyond permeability, such as capillary pressure or structural stability, may influence overall effectiveness in heat pipe wicking.

## 6. Conclusions

This study captured droplet dynamics with less than 6% error between experimental and numerical results, revealing variations in contact angles across TPMS-Gyroid, Octet, Diamond, and Isotruss structures, with Isotruss (2 mm^3^) showing the highest angle. TPMS-Gyroid showed favorable contact angles, suggesting further study potential. Wettability impacted penetration times, with larger unit cells causing faster droplet penetration, highlighting the importance of design parameters in thermal management. Capillary performance tests showed TPMS-Gyroid as the best performer, followed by Octet, Isotruss, and Diamond. Structures with 2 mm^3^ and 3 mm^3^ unit cells showed capillary effects, while 4 mm^3^ structures did not. Permeability tests excluded 4 mm^3^ structures due to poor capillary performance, and 2 mm^3^ structures were omitted due to printing challenges. Among the remaining, Octet had the highest permeability, outperforming Isotruss, Diamond, and TPMS-Gyroid, which showed strong capillary performance but lower permeability, indicating its potential for fluid retention in thermal applications. We validated numerical and experimental results for contact angle and droplet dynamics across porous structures. The study emphasizes structure-dependent wettability and permeability.

The work also addresses practical limitations encountered during fabrication and testing. Specifically, the removal of uncured resin from fine-pore structures proved challenging, and minor variability in droplet placement and image processing introduced measurement uncertainties. Nevertheless, the overall agreement between simulation and experiment with less than 6% error reinforces the reliability of the methods and models applied.

Beyond its immediate findings, this study establishes a foundation for more advanced investigations into phase-change phenomena within porous media. Future work will explore boiling heat transfer in these structures, as well as the fabrication of metallic TPMS lattices for high-temperature and high-performance thermal systems. The insights gained here contribute meaningfully to the broader understanding of how additively manufactured porous architectures can be engineered for next-generation heat pipes, capillary-driven cooling devices, and microfluidic filtration systems.

## Figures and Tables

**Table 1:** Printing parameters for contact angle measurement test.

Structure	Unit Cell (mm^3^)	Beam Thickness (mm)	Porosity	Surface Area (mm^2^)	Volume (mm^3^)	SA/V Ratio (mm^−1^)
TPMS-Gyroid	2	0.479	50.04	72,631.25	11,990.4	6.05
	3	0.721	50.08	49,185.66	11,980.8	4.10
	4	0.958	50.04	37,683.29	11,990.4	3.14
Octet	2	0.444	50.02	79,630.53	11,995.2	6.64
	3	0.656	50.01	55,538.52	11,997.6	4.62
	4	0.859	50.00	43,220.95	12,000.0	3.60
Diamond	2	0.699	50.08	53,578.24	11,980.8	4.47
	3	1.040	50.08	36,659.61	11,980.8	3.05
	4	1.375	50.01	28,269.19	11,997.6	2.35
Isotruss	2	0.500	50.00	72,284.93	12,000.0	6.02
	3	0.740	50.09	50,126.38	11,978.4	4.18
	4	0.969	50.02	39,224.40	11,995.2	3.27

**Table 2:** Printing parameters for capillary rate of rise test and permeability experiment.

Structure	Unit Cell (mm^3^)	Beam Thickness (mm)	Porosity	Surface Area (mm^2^)	Volume (mm^3^)	SA/V Ratio (mm^−1^)
TPMS-Gyroid	2	0.426	50.09	28,383.90	3992.8	7.10
	3	0.603	50.05	20,740.63	3996.0	5.19
	4	0.644	50.08	19,921.61	3993.6	4.98
Octet	2	0.401	50.05	30,873.67	3996.0	7.72
	3	0.565	50.04	22,871.78	3996.8	5.72
	4	0.654	50.00	20,645.43	4000.0	5.16
Diamond	2	0.642	50.03	20,433.54	3997.6	5.11
	3	0.916	50.05	14,782.10	3996.0	3.69
	4	1.067	50.05	13,097.75	3996.0	3.27
Isotruss	2	0.453	50.05	27,787.75	3996.0	6.95
	3	0.639	50.01	20,634.40	3999.2	5.15
	4	0.740	50.02	18,489.23	3998.4	4.62

**Table 3:** The following equations were used by the modeler for creating TPMS structures used in this study.

TPMS Architecture	Equation f(x, y, z) = 0
Fischer–Koch S (FKS)	cos(2x)⋅sin(y)⋅cos(z) + cos(2y)⋅sin(z)⋅cos(x) + cos(2z)⋅sin(x)⋅cos(y)
Gyroid (G)	cos(x)⋅sin(y) + cos(y)⋅sin(z) + cos(z)⋅sin(x)
Schwarz primitive (SP)	cos(x) + cos(y) + cos(z)

**Table 4:** Permeability test results of 3 mm^3^ unit cell structures.

Structure	Permeability, k (m/s)			
	Test-1	Test-2	Test-3	Test-4
Octet	0.0089	0.0088	0.009	0.0090
Isotruss	0.0167	0.0168	0.0166	0.0168
Diamond	0.0184	0.0188	0.0182	0.0175
TPMS-Gyroid	0.0192	0.0181	0.0186	0.0188

## Data Availability

The original contributions presented in this study are included in the article. Further inquiries can be directed to the corresponding author.
